# T-Cell Immunotherapy for Pediatric High-Grade Gliomas: New Insights to Overcoming Therapeutic Challenges

**DOI:** 10.3389/fonc.2021.718030

**Published:** 2021-10-25

**Authors:** Dalia Haydar, Jorge Ibañez-Vega, Giedre Krenciute

**Affiliations:** Department of Bone Marrow Transplantation & Cellular Therapy, St. Jude Children’s Research Hospital, Memphis, TN, United States

**Keywords:** CAR T cells therapy, immunotherapy, tumor antigen, immune tumor microenvironment, pediatric-type diffuse high-grade glioma, childhood CNS tumors

## Abstract

Despite decades of research, pediatric central nervous system (CNS) tumors remain the most debilitating, difficult to treat, and deadliest cancers. Current therapies, including radiation, chemotherapy, and/or surgery, are unable to cure these diseases and are associated with serious adverse effects and long-term impairments. Immunotherapy using chimeric antigen receptor (CAR) T cells has the potential to elucidate therapeutic antitumor immune responses that improve survival without the devastating adverse effects associated with other therapies. Yet, despite the outstanding performance of CAR T cells against hematologic malignancies, they have shown little success targeting brain tumors. This lack of efficacy is due to a scarcity of targetable antigens, interactions with the immune microenvironment, and physical and biological barriers limiting the homing and trafficking of CAR T cells to brain tumors. In this review, we summarize experiences with CAR T–cell therapy for pediatric CNS tumors in preclinical and clinical settings and focus on the current roadblocks and novel strategies to potentially overcome those therapeutic challenges.

## Introduction

Central nervous system (CNS) tumors are second only to leukemias, in terms of being the most common pediatric malignancies, and gliomas account for a quarter of all childhood cancers in the U.S (1). Although the prognosis for pediatric low-grade diffuse gliomas (pLGGs) remains promising, with a probability of 5-year survival above 95%, pediatric high-grade diffuse gliomas (pHGGs) are the deadliest childhood cancers (2). Indeed, 5-year survival drops to less than 10% for patients with pHGGs and 1% for those with diffuse midline glioma, *H3 K27-altered* (DMG; previously known as DIPG) (2). Most advances in therapies for pediatric CNS tumors have relied on experiences from adult brain tumor trials. However, given the developmental and histopathological differences in adult and pediatric diseases, such approaches might not provide the most optimal outcome. Specifically, pHGGs present unique molecular heterogeneity and epigenetic characteristics that render the application of results from adult trials ineffective. Moreover, pHGGs form a niche of tumor cells in distinct brain locations surrounded by tight junctions of the blood brain barrier (BBB) and a complex immune tumor microenvironment (TME) (3). These features affect tumor behavior and the efficacy of new therapeutics. Importantly, the 2021 World Health Organization (WHO) Classification of CNS Tumors (CNS5) recognized two new families of tumor types, “p”LGG and “p”HGG, to reflect on the importance of separating pediatric-type and adult-type gliomas (4). Therefore, when developing new therapies that target pHGGs, we need to consider the distinct characteristic of childhood CNS cancers. In this review, we focus on non-spinal tumors and therefore the term pediatric brain tumors (PBTs) will refer to childhood brain tumors exclusively.

**Figure 1:**
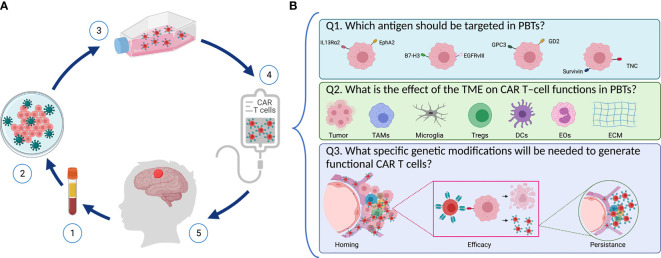
Designing safe, effective, and long-lasting T-cell therapies for PBTs. **(A)** Scheme representing CAR T–cell treatment *via* adoptive T cell transfer in pediatric patients with brain tumors. (1) T cells are isolated from patient’s blood followed by (2) T cell activation and reprograming in the lab to express the chimeric antigen receptor (CAR) using viral vectors. (3) CAR T–cells are then expanded, and they undergo quality control testing (4) prior to infusion into the patient (5). **(B)** Key questions to address when designing CAR T–cells for PBTs. (1) Selecting an appropriate target: Several TAAs (including IL13Rα2, EphA2, B7-H3, GD2, EGFRvIII, and TNC) are expressed in PBTs with heterogenous expression patterns. (2) Overcoming the suppressive immune TME: Immune cells (like TAMs, DCs, Tregs, and EOs) infiltrate PBTs and they induce different immune interactions that affect the CAR T–cells’ ability to perform their cytotoxic properties. (3) Once infused, CAR T–cells need to home to the patient’s tumor and exert their cytolytic activity while expanding and persisting to create long-lasting effects.

Successful therapies for PBTs need to overcome surgical inaccessibility, limited penetration of chemotherapy drugs, inherent resistance to conventional therapies, and long-term adverse effects. Thus, cell-based immunotherapy using chimeric antigen receptor (CAR)–engineered T cells is an exciting alternative for treating debilitating PBTs. The use of CAR T cells to target a tumor relies on engineering and re-directing a patient’s own immune cells (**Figure 1**) to attack tumor cells through selective target recognition and activation of cytotoxic machineries. Activated CAR T cells lyse the tumor expressing the recognized target while sparing normal cells in the absence of target expression. Yet, despite defining several potentially effective targets in adult brain tumors (e.g., IL13Rα2, HER2, and EGFRvIII), clinical testing of CAR T cells in brain tumors failed to produce complete and sustainable antitumor responses (5–8). Challenges included antigen heterogeneity and emergence of antigen-loss variants, limited T-cell persistence, and recruitment of suppressive immune cells in patients (9). Here, we review the lessons learned from preclinical and clinical testing of CAR T cells, with a specific reflection on the unique features of pHGGs that need to be addressed in future efforts to develop effective and safe CAR T–cell immunotherapies for PBTs.

## Pediatric-Type Diffuse High-Grade Glioma

Pediatric gliomas include heterogenous groups of brain malignancies that are histologically similar to adult tumors but with distinct molecular and genetic alterations that dictate clinical behavior and therapeutic considerations (10). Gliomas arise from glial cells, including astrocytes, oligodendrocytes, microglia, or ependymal cells, that normally support neuronal functions. According to the new WHO CNS5 classification, gliomas are classified into two tumor types, pLGG and pHGG, depending on histologic, genetic, and other molecular biomarkers (4). Furthermore, the new classification allows tumor grading within each tumor types (grades 1-4) depending on clinicopathlogical and combined histological and molecular characteristics (4).

The pHGGs are highly aggressive brain tumors with minimal response to standard therapies, including surgery, radiation, and/or chemotherapy. Although less common than pLGGs, pHGGs are the leading cause of death from childhood CNS tumors in the U.S. The pHGGs include four tumor types: Diffuse midline glioma, *H3 K27-altered*; Diffuse hemispheric glioma, *H3 G34-mutant*; Diffuse pediatric-type high-grade glioma, *H3-wildtype* and *IDH-wildtype*; and Infant-type hemispheric glioma (4). Sharing some morphologic features with pLGGs, high-grade tumors are characterized by amplified cell division, increased invasiveness, and augmented neovascularization (10). Additionally, the new CNS5 classification stratifies tumors according to unique genetic and epigenetic alterations which define tumor behavior and response to therapy. For example, patients with isocitrate dehydrogenase (*IDH1* and *IDH2*) mutations tend to have a better prognosis compared to those with *IDH* wild-type tumors (11). Moreover, patients with histone *H3.1* mutations tend to have better overall survival (15 months), while patients with *H3.3* mutations have reduced overall survival (9 months) and enhanced resistance to radiotherapy (12). Therefore, understanding the effects of specific molecular alterations on disease severity and tumor behavior is essential to help select individualized immunotherapeutic targets.

Identifying a patient’s histologic tumor diagnosis is essential to guiding therapeutic approaches; yet, it is not sufficient to guide the development of novel directed therapies. For instance, patients with brain stem tumors may require additional modification of adoptive therapies to enhance the accessibility of the infused products to the tumor, compared to those not located in the brain stem. Alternatively, patients with specific genetic or epigenetic alterations may express unique targets or pose specific treatment challenges that require arming new therapies against such stresses. Therefore, understanding the genetic/epigenetic heterogeneity of pHGGs is also key to stratifying patients into subgroups that will benefit most from specific therapies, rather than into groups that require unique modifications to render cellular therapies more effective and safer.

Of note, while the new CNS5 classification regroups the tumor type previously known as diffuse intrinsic pontine glioma (DIPG) into the diffuse midline glioma, H3 K27-altered (DMG) tumor type; we still refer to DIPGs later in our review when citing previous preclinical studies and clinical literature that did not specify the status of the molecular alterations.

## Establishing Successful CAR T–Cell Therapy for PBTs

Standard therapies have failed to improve the outcome of pHGG and have been associated with long-term debilitating adverse effects. Therefore, adoptive immunotherapy using T cells expressing CARs could offer potentially safer, more specific targeting of PBTs by eliciting directed immune responses against tumor cells, while sparing normal cells that do not express the targeted antigen (13). CARs are synthetic receptors composed of an extracellular tumor-specific antigen-recognition domain (usually a single-chain variable fragment of a monoclonal antibody) connected to a hinge, a transmembrane domain, and intracellular signaling domains (14). Upon recognition of tumor antigens, signaling through CAR domains activates T-cell functions, resulting in tumor cell lysis (14). CAR T cells show potent and sustained antitumor activity in patients with hematologic malignancies, as evidenced by continuous FDA approvals of CD19-CAR T cells for different pediatric and adult B-cell leukemias (15). However, early clinical studies with CAR T cells for adult brain tumors failed to recapitulate the potent anti–brain tumor activity of CAR T cells seen in preclinical testing (14). Thus, the development of CAR T–cell therapies for PBTs will be even more complicated, given the unique patient population.

The following questions must be kept in mind as we design and create safer, effective, and long-lasting T-cell therapies for PBTs (**Figure 1**): First (Q1), which antigen should be targeted in PBTs, and will one target be sufficient for a broad group of pHGGs? Second (Q2), what is the effect of the TME on CAR T–cell functions in PBTs? For instance, tumor cells can exert environmental stress (e.g., hypoxia, inhibitory checkpoint ligands, release of suppressive mediators) on CAR T cells, thereby preventing their activation and cytotoxic functions (16, 17). Moreover, suppression of CAR T cells by infiltrating immune cells within the complex tumor niche represents a hurdle to overcome (18, 19). Lastly (Q3), what specific modifications will be needed to generate functional CAR T cells in PBTs? To generate the desired therapeutic efficacy, CAR T cells need to home to the tumors, eradicate tumor cells without on-target off-tumor toxicities, and persist to resolve any recurrent tumors? In the following sections, we will define some aspects of pHGGs that are essential for developing effective adoptive T-cell therapies for children.

### Q1. Which Antigen Should Be Targeted in PBTs?

CAR T cells are engineered to selectively recognize and target tumor-associated antigens (TAAs) expressed on tumor cells (20). Targetable TAAs are characterized by exclusive expression on tumor cells, with minimal expression on normal tissues to prevent on-target off-tumor toxicities (21). However, with a limited number of tumor-specific antigens exclusively expressed on tumor cells, targeting TAA that are present on normal cells must consider strategies to prevent, reverse, or manage any potential toxicities that result from lysing normal cells expressing the selected target (discussed in later sections). Traditional targets focus on surface proteins, but novel strategies for target discovery expand to include posttranslationally modified proteins, carbohydrate repeats, lipids, glycoproteins, and alternative splice variants (21). Brain tumor–specific CAR T–cell targets have been identified, mostly based on samples from adult brain tumors; however, a limited number of studies have evaluated their validity in pediatric populations (22–24). Some studies have shown that antigen expression in PBTs does not recapitulate the exact patterns seen in adult brain tumors. Moreover, a study by our group using PBT xenografts revealed a unique inter- and intra-patient variability when TAA expression was compared across tumor subtypes (22). Additionally, experiences from preclinical and clinical studies in adults suggest that it is unlikely that one target will be sufficient to cure heterogenous brain tumors (21). Thus, identifying selective targets that are effective and safe for patients with heterogenous PBTs is a key question for the development of successful CAR T–cell therapies in pHGGs. Here we summarize and review the current knowledge on potential CAR targets in pHGGs.

#### Disialoganglioside

Disialoganglioside (GD2) belongs to the glycosphingolipid family of gangliosides expressed on outer plasma membranes of various cell types (25). Gangliosides regulate cell interaction, adhesion, and signal transduction (25). GD2 is widely expressed on different solid tumors (neuroblastoma, melanoma, osteosarcoma) but has limited expression on normal cells, including nerves, lymphocytes, and melanocytes (25). Xenografts of pHGGs robustly express GD2 (~84%) (22). Moreover, GD2 is uniformly expressed in DMGs (24). The use of GD2 as immunotherapeutic target showed potent antitumor effects but was associated with unwanted adverse effects (including neuropathic pain and headaches) due to limited expression in normal tissues (26). Similarly, anti-GD2 CAR T cells have shown potent antiglioma efficacy in preclinical DMG models but resulted in hydrocephalous (24). Nevertheless. Currently, two ongoing clinical trials are evaluating GD2-CAR T cells in pediatric patients with DMG or other pHGGs (NCT04196413 and NCT04099797, **Table 1**) (31). Early results from patients with DMGs treated with GD2 CAR T cells suggest promising clinical responses along with tolerable safety profiles (including incidences of manageable cytokine release syndrome and neurotoxicity) (28). Therefore, data from these clinical studies will define the strategies for GD2-directed CAR T–cell therapy and provide general insights on CAR T–cell therapy efficacy and safety in pHGGs.

**Table 1:** Summary of ongoing clinical studies with CAR T cells for PBTs.

NCT Number	Target	Delivery	Age	Study Results	Toxicity
NCT04510051	IL13Rα2	IT	4 Years to 25 Years	No Results Available	No Results Available
NCT04185038	B7-H3	IT, IC	1 Year to 26 Years	Stable clinical disease with detectable CAR T cells in CSF (27)	No DLTs (27)
NCT03638167	EGFR	IT, IC	1 Year to 26 Years	No Results Available	No Results Available
NCT04099797	GD2	IV	12 Months to 18 Years	No Results Available	No Results Available
NCT04196413	GD2	IV	2 Years to 30 Years	Durable clinical responses and marked CAR T cell expansion (28)	CRS (Grade 1-3) ICANS (Grade 1-2) TIAN No other DLTs (28)
NCT03500991	HER2	IT, IC	1 Year to 26 Years	Clinical and laboratory evidence of local CNS immune activation (29)	No DLTs (29)
NCT02442297	HER2	IT, IC	3 Years and older	No Results Available	No Results Available
NCT01109095	HER2	IV	Child, Adult, Older Adult	1/16 partial response, 7/16 stable disease (6, 30)	No DLTs (6, 30)

(IV, Intravenous; IT, Intrathecal/ventricular; IC, Intratumor/cavity; DLT, Dose limiting toxicity; CRS, Cytokine release syndrome; TIAN, Tumor Inflammation-Associated Neurotoxicity; ICANS, Immune effector cell-associated neurotoxicity syndrome).

#### B7 Homolog 3

B7 homolog 3 (B7-H3), also known as cluster of differentiation 276 (CD276), is a member of the B7 and CD28 immune checkpoint family (32). B7-H3 is expressed on peripheral lymphoid tissues and antigen-presenting cells and has a controversial role in immune stimulation and inhibition (including promoting T-cell cytotoxicity *vs* inhibiting T-cell proliferation and activation) (33). B7-H3 expression on solid or hematologic malignancies is associated with reduced survival and enhanced cancer progression through mechanisms dependent on immune evasion, enhanced macrophage recruitment, and elevated levels of suppressive cytokines (32, 33). B7-H3 is highly expressed in pHGG (~100%), with its highest expression intensities in more aggressive tumors, like DMG (22, 23, 34). B7-H3–directed CAR T cells showed potent antiglioma efficacy in preclinical xenograft and syngeneic models (22, 35, 36). Although B7-H3 is expressed at some level on normal tissues, including the adrenal gland, salivary gland, and gastric epithelial cells (37–39), preclinical studies show a favorable B7-H3–CAR T–cell safety profile (22, 38). Ongoing clinical trials that are evaluating anti–B7-H3 CAR T cells in adult GBM (NCT04077866) and in pHGG and DMG (NCT04185038, **Table 1**). Preliminary results from pediatric patients with CNS tumors show that serial doses of B7-H3 CAR T cells result in clinically stable disease in the absence of any dose limiting toxicities (27). Thus, data from ongoing clinical studies will further characterize the safety and efficacy of this CAR target.

#### Interleukin-13 Receptor Alpha 2

Interleukin-13 receptor alpha 2 (IL13Rα2) is a subunit of the IL13 receptor complex. Closely related to the α1 subunit, IL13Rα2 is thought to function as a decoy receptor, reversing IL13-mediated JAK/STAT signaling transduction (40). IL13Rα2 is overexpressed in various solid tumors, including breast, prostate, and pancreatic cancer, with minimal expression on normal tissues (e.g., spermatocytes) (41). IL13Rα2 expression is associated with enhanced metastasis, invasiveness, and reduced survival (41). In gliomas, the level of IL13Rα2 expression increases with malignancy grade, with higher expression in grades III and IV (53%-73%) (42). IL13Rα2 is overexpressed in PBTs (~68%), including pHGGs (22). CAR T cells targeting IL13Rα2 have shown potent antiglioma activity and enhanced survival in preclinical and clinical studies. An ongoing trial is assessing the efficacy of IL3Rα2-CAR T cells in children with refractory glioma (NCT04510051, **Table 1**) (8, 43). Given its favorable safety profile but highly heterogenous expression profile in PBTs, IL13Rα2 will most likely be a promising target for dual-targeting regimens or for specific populations that are resistant to other robustly expressed TAAs.

#### Human Epidermal Growth Factor Receptor 2

Human epidermal growth factor receptor 2 (HER2; also known as ErbB2), is a transmembrane receptor tyrosine kinase (44). The role of HER2 in tumorigenesis was first defined in breast cancer, where HER2-mediated signaling transduction drives cell proliferation, invasion, survival, and metastasis (45). HER2 expression is inversely correlated with survival and mediates faster tumor growth, increased metastatic potential, increased disease grade, and enhanced resistance to endocrine therapies (45). HER2 is robustly expressed in gastric, ovarian, prostate, and CNS tumors (44). Specifically, HER2 is highly expressed in adult HGG (~42%) and pediatric medulloblastomas (~40%), along with less robust expression in pHGGs (~37%) (22, 46). HER-2–directed CAR T cells have shown potent antiglioma efficacy in preclinical and clinical studies (6, 47). Although HER2-CAR T cells have been well tolerated so far, toxic side effects associated with HER2-directed therapies (trastuzumab) have been observed (30, 48, 49). Additionally, ongoing trials in pHGGs are evaluating HER2-CAR T cells in refractory disease (NCT03500991, **Table 1**) and early results suggest that repeated locoregional delivery of HER2 CAR T cells are well tolerated in these young patients (29). Collectively, heterogenous expression of HER2 in normal tissues and in pHGGs necessitates close evaluation of HER2 as a CAR target for PBTs. If selected as a target, the incorporation of a safety switch in the CAR design needs to be considered to avoid any unintended adverse events.

#### Ephrin Type-A Receptor 2

Ephrin type-A receptor 2 (EphA2) belongs to the ephrin class of receptor tyrosine kinases (50). Upon interaction with its ligand, ephrin A1, EphA2 engages in bidirectional signaling that controls cell adhesion, motility, and tissue development (51). In normal tissues, EphA2 is upregulated and expressed only in rapidly proliferating cells (50). However, in several cancers (i.e., lung, prostate, breast, and brain tumors), EphA2 is robustly and highly expressed (51). A recent study by our group shows that EphA2 is expressed in about 28% of patient-derived xenografts of pHGGs (22). EphA2 overexpression results in extracellular matrix deposition, enhanced proliferation, invasiveness, and angiogenesis (52), thus resulting in reduced survival, increased metastasis, and enhanced malignant progression (51). EphA2-directed CAR T cells have shown promising antiglioma activity in preclinical brain tumor models (53, 54), and at least one clinical trial is accruing patients with recurrent gliomas to evaluate the safety and efficacy of EphA2-CAR T cells (NCT03423992). Due to its role in tumor progression and invasiveness and its potentially safe profile with limited expression on normal tissue, EphA2 is a promising target for CAR T–cell immunotherapy of PBTs that demands further clinical and preclinical investigation.

#### Epidermal Growth Factor Receptor Splice Variant III

Epidermal growth factor receptors comprise a family of receptor tyrosine kinases (55). Overexpression or mutation of these receptors is a negative prognostic factor in several solid tumors, including lung, breast, ovarian, and CNS cancers (56). Epidermal growth factor receptor splice variant III (EGFRvIII) is the most common EGFR mutation in pHGG resulting from a fusion of exon 1 to exon 8, thereby triggering aberrant ligand-independent receptor activation (57, 58). In PBTs, EGFRvIII is overexpressed in pHGGs (14%-40%) (58, 59). Due to its lack of expression in normal tissues, EGFRvIII is considered an ideal CAR target (60). However, EGFRvIII-directed CAR T cells showed minimal antitumor activity in adults with glioma (NCT02209376) (7). Interestingly, a recent study demonstrated that EGFRvIII, due to its tumor-specific expression, can be successfully used in a SynNotch-CAR system, where it is responsible for turning on the expression of a dual-antigen–targeting CAR (IL13Rα2 and EphA2) at a tumor site. This approach led to a less exhausted CAR T–cell phenotype and improved anti-GBM activity *in vitro* and *in vivo* (61). This study emphasizes the need to incorporate novel methods to target antigens expressed at lower intensities on tumor cells. Moreover, targeting EGFRvIII also shows promising results in vaccine trials in DMG (NCT01058850) (62) while a new trial will be evaluating EGFR CAR T cells in pediatric patients with refractory CNS tumors (NCT03638167, **Table 1**). Therefore, given all the clinical and preclinical data, EGFRvIII is most likely a promising target for immunotherapy of PBTs that may require additional CAR modifications or dual targeting approaches (62).

#### Tenascin-C

Tenascin-C (TNC) is an embryonic glycoprotein expressed on neurons and astrocytes that functions as an adhesion-modulating protein (63). TNC undergoes posttranslational modification (alternative splicing), which allows the protein to interact with fibronectin and several other growth factors, thus inducing a wide range of functions related to focal adhesion, matrix formation, and cell motility (64). Alternatively spliced TNC is minimally expressed in normal tissues but robustly upregulated in tumors and extracellular matrices of breast, lung, kidney, prostate, and CNS tumors (64). Expression of TNC splice variants is associated with poor prognosis and enhanced tumor invasiveness and metastatic potential (64, 65). TNC is highly expressed in adult HGGs (85%-96%) and pHGGs (>42%) (66, 67). TNC expression in DIPGs correlates with higher tumor grade and more frequent *H3K27M* mutation (67). TNC-targeting immunotherapy, including monoclonal antibodies, therapeutic vaccines, and antibody–drug conjugates, have shown promise in preclinical and clinical studies in CNS tumors and other tumor models (NCT01131364, NCT01134250) (68). Thus, TNC-targeting T-cell therapies are promising not only as a tumor-targeting approach but also as a target that can potentially enhance CAR T–cell permeability and delivery to brain tumors, with potential targeting of the extracellular matrix and TME.

#### Other Potential Targets

##### Survivin

As an inhibitor-of-apoptosis protein, survivin regulates programmed cell death and cell cycle progression (69). Survivin is expressed during embryonic development but is absent in normal terminally differentiated tissues (70). It is highly expressed in primary and secondary adult GBMs (83% and 46%, respectively) and in pHGGs and medulloblastoma (71–74). Survivin-targeted adoptive T-cell products have potent anti–acute myeloid leukemia activity, and other survivin-based vaccines, cellular therapies, and gene therapies have shown potent antitumor efficacy and favorable safety (71, 75, 76). Therefore, survivin is an ideal target for cancer immunotherapy due to its limited expression on normal cells and wide expression on PBTs (69, 70).

##### Glycoprotein 100

Also known as PMEL17, glycoprotein 100 (Gp100) is a premelanosomal protein expressed in melanocytes (77). It is involved in melanosome development, including vesicular formation, structural maturation, and pigmentation (78). Gp100 is more robustly expressed in adult HGGs (>80%) than in pHGGs (~46%) (79). The combination of a Gp100-directed vaccine and IL2 showed promising enhanced survival in patients with melanoma (80). Additionally, dual-specific T cells engineered with Her2-directed CAR and Gp100-specific T-cell receptor repertoire showed durable responses in murine solid tumor models, and transgenic T cells directed against Gp100 showed significant survival advantage in preclinical DIPG models (81, 82). Thus, Gp100 is another target that should be considered for dual-targeting products in specific PBT populations.

##### Glypican-3

A heparan sulfate proteoglycan, glypican-3 (GPC-3) is attached to the cell surface by a glycosyl–phosphatidylinositol anchor (83). GPC-3 is expressed in fetal lung, liver, and kidney tissues during embryonal development and is very minimally expressed in normal adult cells (84). It is also involved in tumorigenesis of embryonal and pediatric tumors due to its role in malignant transformation *via* Wnt/β-catenin–, Hedgehog-, and FGF-signaling alterations (84). GPC-3 is overexpressed in pHGGs and pLGGs (85). GPC-3–targeted CAR T cells in murine models of hepatocellular carcinoma showed potent antitumor activity without any significant toxicities, while also targeting soluble GPC-3 antigens (86, 87). Thus, anti-GPC-3 CAR T cells could potentially recognize GPC-3–expressing gliomas and GPC-3 antigens shed in the TME, which poses another target for glioma extracellular matrices that could be useful for dual CAR-targeting strategies.

##### Neogenin

As part of the immunoglobulin superfamily of receptors involved in cell–cell interactions neogenin is normally expressed during embryogenesis and is essential for axonal navigation and adult neurogenesis (88). Neogenin and its ligand, netrin-1, are highly expressed in solid and CNS tumors, including medulloblastoma and glioma (89, 90). In pHGGs, netrin-1 overexpression mediates enhanced oncogenic astrocyte migration, tumor invasion, and metastasis (91). Neogenin is also highly expressed in DMG, where it drives tumor invasiveness and worsens prognosis (92). Neogenin-targeting monoclonal antibodies reverse its tumorigenic effects in DMG models (92). Therefore, neogenin holds great promise as a novel CAR target to reduce tumor burden and invasive tumor phenotypes.

In summary, the TAAs described above have been extensively studied as targets for immunotherapy and have unique characteristics that could serve as successful targets for CAR T–cell therapy in PBTs. Expression patterns in PBTs are mostly heterogenous with some TAAs more robustly expressed across tumor subtypes (GD2, B7-H3, IL13Rα2), while other targets have variable expression frequencies and intensities (HER2, EphA2, EGFRvIII). Here we described the most common, well-studied TAAs, though other potential PBT-specific molecules, such as survivin (75), Gp100 (93), GPC-3 (83), and neogenin (92), should also be considered as CAR T–cell targets for PBTs. Moreover, TAAs like GPC-3 and neogenin have the potential to target the TME and extracellular matrices; this property could be exploited to enhance delivery and accessibility of infused CAR T–cell products. Other unique targets, like Gp100 and survivin, have potential use in multiantigen CAR-targeting approaches. Although expression profiles for most of these TAAs are well defined in PBTs, their validation as targets for CAR T–cell therapy is warranted. **Table 1** provides a summary for ongoing CAR T cell clinical trials in PBT patients (extended details on each trial are available in **Supplemental Table 1**). Preclinical testing in representative PBT models and clinical testing in specific pediatric patient populations should guide target selection and CAR designs to achieve the desired therapeutic benefits. Moreover, development of effective CAR T–cell therapies require additional screening and novel target discovery and validation in PBTs.

### Q2. What Is the Effect of the TME on CAR T–Cell Functions in PBTs?

**Figure 2:**
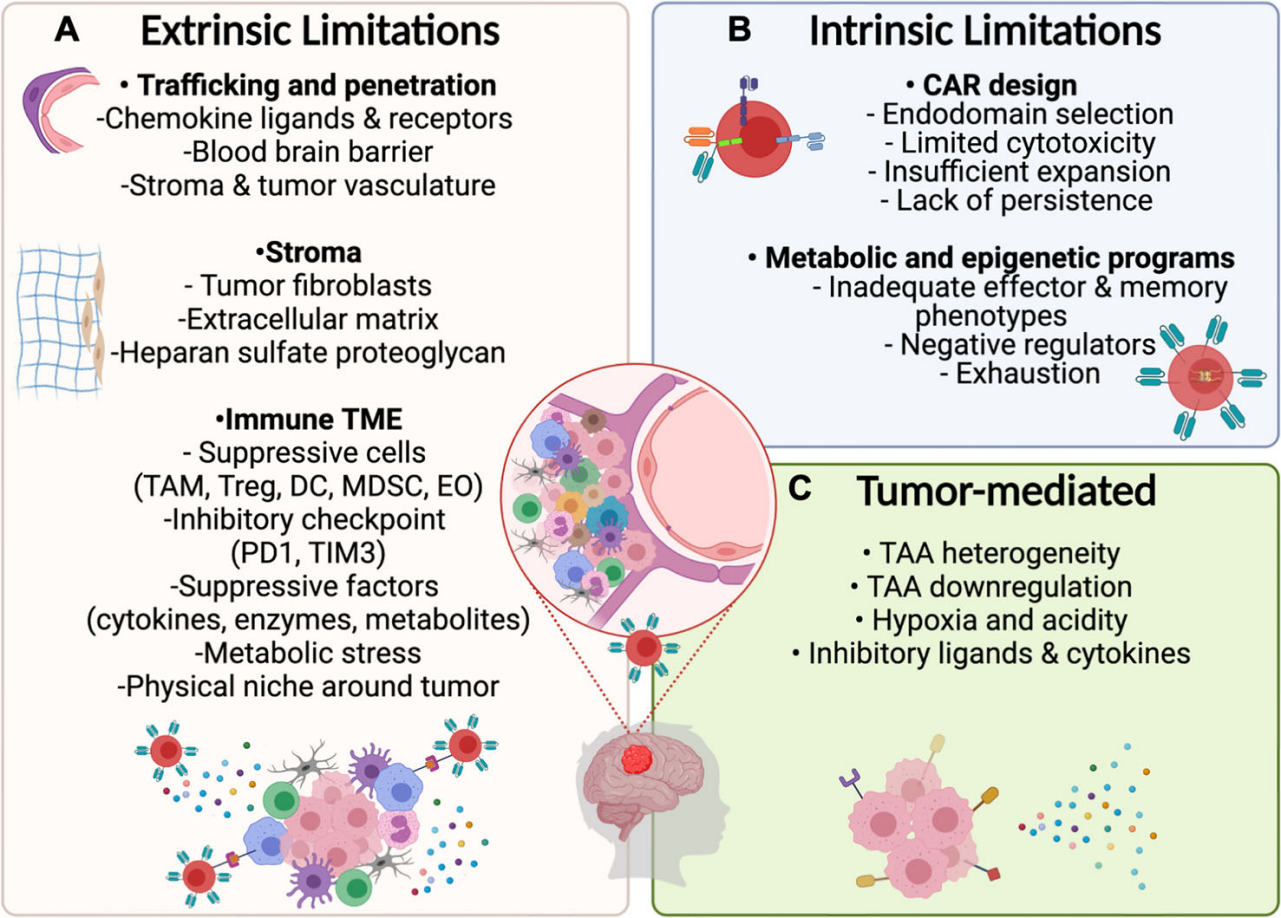
Potential limitations for CAR T–cell therapy in PBTs. **(A)** Extrinsic Challenges for CAR T–cell immunotherapy will depend on the ability of designed products to home to the tumor by overcoming the physical limitations induced by the BBB and stroma surrounding the tumor followed by surviving the suppressive immune TME including inhibitory cytokines and ligands. **(B)** Intrinsic limitations depend on optimizing the CAR design and programs that control metabolic and epigenetic functions to mediate necessary cytotoxic mechanisms while preventing exhaustion. **(C)** Tumor cells may resist CAR T cell therapies by downregulating targeted antigens and by exerting environmental stress on CAR T–cells through the release of suppressive cytokines and expression of inhibitory ligands.

During the past decade, it has been well established that the TME limits CAR T–cell trafficking to tumors and suppresses their effector functions through direct physical contact or molecular interactions (**Figure 2**). Complex tumor vasculature, tumor-induced suppression of chemokine ligands, and reduced expression of chemokine receptors on CAR T cells limit their migration to the tumor (94, 95). Additionally, the deposition of extracellular matrix and accumulation of cancer-associated fibroblasts hinder CAR T–cell penetration and mediate immunosuppression (19, 94). Finally, disrupted BBB permeability and altered endothelial cell functions in pHGGs can affect the accessibility and trafficking of adoptive cell products to the targeted tumor cells (96).

In the brain, the TME includes a highly specialized immunologic niche called the immune TME. In pHGGs, the immune TME contains suppressive immune cells, like tumor-associated neutrophils, myeloid-derived suppressor cells, dendritic cells (DCs), and regulatory T cells (Tregs) (18). Expression of inhibitory checkpoint ligands (TIM3, PD1) on these cells suppresses T-cell proliferation and cytokine release (97). Additionally, the release of inhibitory mediators and metabolites (TGFβ, IL-10, IDO-1) blocks T-cell functions and further recruits other suppressive immune cells (18, 19). In this section of the review, we highlight the specific features of the immune TME in pHGGs and some unique features of DMG that are key to developing successful CAR T–cell therapies in PBTs.

#### The Immune TME in pHGGs

The TME in pHGGs is heterogenous, with different immune cell compositions at each tumor grade or genetic classification (98). Immune TME heterogeneity depends on the plasticity and distinct immunomodulatory functions of tumor-associated macrophages (TAMs), Tregs, DCs, eosinophils (EOs), and other suppressive immune cells and mediators.

#### Tumor-Associated Macrophages

Macrophages are key components of the immune TME. TAMs represent more than 30% of nonmalignant cells in adult HGGs, and they are negatively correlated with immune escape, resistance to immune checkpoint blockade, and disease progression (99). However, increased TAM frequencies in pHGGs does not directly correlate with poor outcomes (100). Specifically, two subsets of TAMs are present in pHGGs and are derived from either embryonic microglia or tumor-infiltrating monocytes with distinct functions and effects on antitumor immune responses (98, 101). Microglia-derived TAMs are located around the tumor edges and upregulate inflammatory, metabolic, and suppressive cascades, while monocyte-derived TAMs are generally located within the tumor; they upregulate genes associated with cell proliferation, motility, and migration (102). Distinct TAM subsets in pHGGs play different roles in promoting tumor growth and mediating an immunosuppressive state. Thus, enrichment of monocyte-derived TAMs in pHGGs is associated with poor prognosis and reduced survival (100). Abundance of suppressive monocyte-derived TAMs in pHGGs can potentially limit CAR T–cell therapy by suppressing effector functions (Treg polarization, inducing metabolic T-cell hyporesponsiveness, and direct suppression *via* PD1/PDL1 interaction) and reducing T-cell penetration and chemotaxis to the tumors by forming a chemically and physically suppressive niche around the tumor (103–105). Alternatively, microglia-derived TAMs are most likely essential to promote CAR T–cell effector functions and support their persistence.

#### Regulatory T Cells

The pHGGs are heavily infiltrated with Tregs, constituting almost 15% of non-neoplastic cells (106). Higher frequencies of Treg infiltration in pHGGs correlate with poor overall survival and greater WHO disease grade (106). Besides suppressing endogenous antiglioma T-cell responses, Tregs also promote tumor growth by inducing STAT3-mediated hypoxia (107). Tregs in pHGGs are heterogenous, with most cells being thymus-derived *versus* another native population induced and maintained by suppressive factors within the glioma TME (98). Tregs in pHGGs also upregulate proapoptotic genes (*Bax*, *Bak*, *Bim*), thus promoting their fitness and survival within the glioma TME, augmenting tumor growth, and suppressing T-cell effector molecules (e.g., granzyme B) (108). Therefore, Tregs are most likely detrimental for CAR T–cell functions in PBTs, and suppressive mediators may polarize infused CAR T cells into Treg-like phenotypes, thus limiting their cytotoxic functions.

#### Other Immune Cell Populations

##### Dendritic Cells

Multiple DC populations infiltrate solid tumors and influence antitumor immunity and response to therapy (98). Increased DC recruitment in preclinical glioma models potentiates CD8+ T–cell responses, as well as response to immune checkpoint blockade (109). Moreover, recent studies suggest that increased DCs in adult HGGs is correlated with worse clinical manifestations without significant contribution to disease grade (110). Although limited data are available on the specific role of DCs in pHGGs, reports suggest that their functions are impaired by tumor-mediated immunosuppression (110). Thus, specific interactions of DCs within the TME in pHGGs need to be further investigated, especially in the context of CAR T–cell therapies, which do not require antigen presentation by DCs but could be suppressed by altered DC functions.

##### Eosinophils

Recruitment of EOs into the glioma TME has been implicated in tumorigenesis and suppression of antitumor immune responses (111). Marked eosinophilia has been observed in patients with HGG, and it correlates with worse prognosis and decreased response to therapy (111). Conversely, correlation analysis in patients with HGGs has shown that lower EO counts are inversely correlated with disease grade and pathology (112). In pHGGs, EOs represent about 13% of noncancer cells, and they tend to cluster with PBTs that are rich in Tregs and natural killer cells (106). Given the plasticity and abundance of EOs, investigating their interaction with CAR T–cell therapies in PBTs is warranted, especially in the context of preconditioning therapies and/or radiation and chemotherapy, which could drastically affect these cells.

#### The Immune TME in DMGs

A limited number of studies have characterized the TME of DMGs, because deciphering this TME is challenging due to the location and diffuse, infiltrative nature of DMGs. Two recent studies of samples obtained at diagnosis or autopsy have reported some preliminary findings on the immune profile of DMGs (113, 114). Both studies reported a CD45+ leucocyte compartment consisting primarily of CD11b+ macrophages with very few CD3+ T cells in primary DMG tissue samples. In addition, a study by Lieberman et al. showed no increase in immunosuppressive CD163+ tumor-associated macrophages in DMG samples when compared to nontumor controls (114). Finally, DMG-derived cell cultures produce markedly fewer cytokines and chemokines than do adult glioblastomas, indicating that DMGs have a noninflammatory phenotype (113). Thus, both studies concluded that DMGs are immunologically “cold” tumors. In addition, a very recent study using a deconvolution approach (methylCIBERSORT) to assess genome-wide DNA-methylation data from pediatric CNS tumors reported that Tregs, EOs, and monocytes infiltrate DMGs (106). Although limited studies are available, they are admirable first approaches trying to illuminate the DMG TME and then apply this knowledge to cellular therapies. More studies are urgently needed to answer the remaining questions, such as how the quiescent DMG immune TME affects CAR T–cell therapy, and if and how do CAR T cells re-shape the immune landscape of DMGs? Given the recent increase in the generation and availability of syngeneic DMG mouse models, the hope is that more studies will be published in the upcoming years, as researchers will be less dependent on patient sample availability. However, some key finding will have to be validated in the clinical setting.

### Q3. What Specific Modifications Will Be Needed to Generate Functional CAR T Cells in PBTs?

Besides selecting targetable TAAs and armoring CAR T cells against the suppressive immune TME, three other T cells-specific functional limitation will determine whether CAR T–cell therapies in PBTs are successful: the ability of infused CAR T–cell products to 1) home to the tumor, 2) exert potent but safe antitumor responses, and 3) establish persistent memory T cells for efficient tumor control. These limitations can be addressed by engineering CAR T cells with additional genetic modifications. In this section, we review CAR-specific modifications and their potential for successful CAR T–cell immunotherapy in pHGGs.

#### Homing

Homing of CAR T cells to CNS tumors depends on their ability to cross the brain parenchyma and utilize chemotactic factors to migrate to the tumor. For a long time, the brain was considered an immune-privileged organ, with tight junctions of the BBB limiting access of immune cells and mediators (115). However, pHGGs are characterized by leaky and fragile vasculature, altered BBB integrity, and reduced expression of essential chemokine ligands and receptors, including CXCL9, CXCL10, CCL2, and CCL12 (116, 117). Thus, during the development of effective CAR T–cell therapies for PBTs, we must consider the need for chemokine ligands and adhesion receptors essential for T-cell trafficking to the brain as well as considering strategies to bypass the physical barriers for delivery of adoptive cell therapy products.

Routes of CAR T cell administration must be carefully considered to ensure appropriate homing of infused cells to the tumors without unwanted adverse effects. CAR T cells can be administered (i) *via* the blood through intravenous (IV) delivery, (ii) *via* the CSF through intrathecal/ventricular (IT) delivery, or (iii) *via* direct delivery to the tumor through intratumor/cavity (IC) injections (118). While preclinical studies comparing different routes of administration suggest that superior anti-tumor efficacy is observed with locoregional delivery (22, 119, 120), clinical experiences show that the three routes of administration can produce desirable therapeutic efficacy (6, 8, 24, 28). With the lack of clinical studies directly comparing different routes of administration in pediatric patients, selecting the most optimal delivery method will depend on feasibility as well as safety considerations. IV administration would be best for targeting tumors that are anatomically challenging (tumor location complicating catheter implantation for IT or IC delivery) or in instances of abnormal CSF flow (inadequate delivery to bulky or parenchymal tumors with IT administration) (118, 121). Alternatively, IT and IC delivery would be best for CAR T cells with limited peripheral activation or trafficking potential where evidence suggests that T cell activation enhances T cell migration to the CNS (14, 122). Additionally, locoregional delivery (IC) should be considered for targeting antigens that are more readily expressed on normal cells to avoid the potential for on-target off-tumor toxicities that may otherwise be pronounced with systemic IV delivery. Lastly, while IV routes of administration may result in systemic toxicities (cytokine release syndrome (CRS) or immune effector cell-associated neurotoxicity syndrome (ICANS)), safety concerns with locoregional delivery necessitate careful considerations for potential inflammation and swelling at the tumor site that may complicate catheter functionality as well as increased risks of infections with these devices (118, 123–126). Therefore, locoregional delivery may be a strategy to enhance homing of CAR T cells through bypassing the BBB; yet, it should be carefully evaluated for specific targets in pediatric patients where CNS tumors may have different anatomical locations and distribution compared to adult tumors (127).

Alternatively, strategies to enhance CAR T cell homing to the tumors in PBT patients may include combination therapies or specific genetic modifications of CAR T cells. For example, using MRI-guided focused ultrasound can potentiate CAR T–cell efficacy and homing by transiently disrupting the BBB and blood–tumor barrier (128–130). Additionally, engineering CAR T cells to express chemokine receptors or utilizing receptors that are endogenously expressed in pHGGs could enhance delivery and penetration of adoptive products. For example, anti-CD70 CAR T cells expressing CXCR1 and CXCR2 traffic better to the brain (95). Such strategies have not been tested in pHGGs, thus determining whether these modifications will be beneficial in PBTs necessitates further preclinical and clinical evaluation.

#### Efficacy and Safety

Promoting the efficacy of CAR T cells, while ensuring the safety of this approach is a key aspect of a successful CAR T–cell therapy. It is now well established that T cells engineered only with a CAR do not produce a sustained antitumor response. Thus, additional genetic modifications must be considered. So far, only a handful of genetic modifications have been tested in adult brain tumor models, let alone PBT models. For example, CARs engineered to express transgenic cytokines, such as IL12, IL15, IL18, have enhanced efficacy in preclinical glioma models (43, 131). Additionally, EphA2-CAR T cells expressing constitutively active IL-7 cytokine receptor (C7R) have enhanced antiglioma efficacy in preclinical models (132).

Engineering tools can also be used to convert tumor-induced suppression of CAR T cells into a beneficial stimulus. For example, engineering CAR T cells to express costimulatory PD1 receptors modified to fuse the extracellular domain of PD1 with an intracellular CD28-activation domain can hijack the system and protect CAR T cells against PD1/PDL1-mediated exhaustion and suppression (133). Other switch receptors, like dominant-negative TGFβ receptor, prevents the otherwise suppressive effects of TGFβ, thus enhancing CAR T–cell cytotoxicity and promoting persistence (134). However, none of these strategies have been evaluated in adult brain tumors or PBTs. Regarding the safety of CAR T cells, every genetic modification has the potential to induce unintended adverse events or uncontrollable T-cell proliferation. Therefore, safety switches, such as CD20, iCas9, tNGFR (135–138), must be considered when designing effective T-cell therapies for PBTs.

Targeting multiple antigens and arming CAR T cells against inhibitory ligands in the TME can also improve their efficacy. Targeting more than one TAA can help overcome the limitations of heterogenous antigen expression and tumor-induced downregulation of targeted antigens (139). Several CAR regimens to target multiple TAAs are available, including the use of pooled products (e.g., combining single-antigen targeting CAR T–cell products), the use of bispecific and trivalent CARs (one T cell expresses several CARs), or the use of tandem CARs (expressing one CAR construct that merges several antigen-recognition domains into one backbone sharing one activation domain) (139, 140). Preclinical studies using CAR T cells against HER2, IL13Rα2, and EphA2 in pooled, bispecific, trivalent, and tandem designs have shown superior antiglioma efficacy (47, 141, 142). Finally, engineering logic-gated (AND, OR, NOT) CAR T cells may offer enhanced specificity and efficacy along with reduced on-target off-tumor toxicities (139). While “OR” logic-gated CAR T cells contain tandem or multiple CAR constructs, a single TAA-CAR interaction is sufficient to activate tumor killing mechanisms which is particularly useful for TAAs with heterogenous expression and to protect against antigen escape (143). Alternatively, “AND” or “NOT” logic-gated CAR T cells protect against on-target off-tumor toxicities by restricting T cell activation to instances where two cognate TAAs are co-expressed (AND); by conditional expression of a second CAR through a SynNotch receptor regulated transcriptional manner (AND); or by selectively killing tumor cells that lack a specific inhibitory ligand which would otherwise suppress the T cells when expressed on normal cells (NOT) (139, 142). However, none of these designs have been evaluated in clinical studies for brain tumors. Although they have the potential for potent synergism and can protect against antigen escape and emergence of antigen-negative tumors, their efficacy and safety in PBTs will depend on how they interact and function in the complex inflammatory pHGGs. If clinical experiences show that pHGGs induce CAR T–cell exhaustion through TME-induced stress, then using pooled products will probably function best to prevent continuous activation of one T cell expressing CARs that target several TAAs at once.

#### Persistence

Lastly, ensuring that CAR T cells persist in patients long enough to control the primary and any recurrent tumors should be carefully considered when designing CAR T–cell therapies for PBTs. T cells exist in multiple differentiated phenotypes (naïve (T_N_), effector (T_EFF_), central memory (T_CM_), stem-like memory (T_SCM_), or tissue resident memory (T_RM_)) (144). Generating CAR T cell products using different pools of T cell differentiation states may be a powerful tool for enhancing self-renewal and persistence. While T_EFF_ cells produce potent cytotoxic responses, they are short-lived compared to T_N_, T_CM_, and T_SCM_ cells (144, 145). The T_N_ cells circulate without being committed to an effector or memory phenotype while T_CM_ and T_SCM_ cells are long-lived and exhibit self-renewal and multipotent differentiation properties (144, 145). Therefore, generating CAR T cell products using less differentiated pools of T_N_, T_CM_, or T_SCM_ cells may be a preferred strategy to enhance persistence and long-lived immunological memory. For instance, a Phase I clinical study using T_CM_-derived CD19 CAR T cells showed improved expansion in leukemia patients (146). However, T_CM_ and T_SCM_ cells express adhesion molecules which favor their homing to lymphoid organs instead of peripheral tissues (147). Since CAR T cells for PBTs need to home to the brain, thorough preclinical and clinical studies need to closely evaluate the use of less differentiated T cells and their potential impact on homing and anti-brain tumor activity. Importantly, T_RM_ cells are tissue-specific memory cells with pluripotent and self-renewal properties similar to T_CM_ and T_SCM_ cells (144, 148). However, isolating these cells for brain tumor patients may not be practical. Moreover, T_CM_ and T_SCM_ cells constitute less than 5% of peripherally circulating cells which would not be sufficient for generating CAR T cell products. Therefore, strategies to enrich for less differentiated T cell phenotypes include using small molecules (Wnt signaling agonists or Akt signaling inhibitors) (149–151), cytokines (IL-21, IL-7 and IL-15) (152, 153), or through transgenic expression of homing ligands and cytokines (154). While some of these modifications have been tested in preclinical models of brain tumors, further clinical evaluation of less differentiated CAR T cell products for patients with brain tumors are needed.

Lastly, enhancing CAR T cell persistence can be achieved through genetic modifications and/or combination therapies. The use of CRISPR/Cas9 technology can help knockout negative T-cell regulators. For instance, silencing PD1 enhances EGFRvIII CAR T–cell activity in preclinical glioma models (155). In addition, knocking-out epigenetic modifier DNMT3A improved IL13Ra2-CAR T cell effector functions in preclinical brain tumor models while antigen negative relapsed have been observed (156). Similarly, using small-molecule inhibitors that can reshape the TME could arm CAR T cells against immune suppression. For example, using an inhibitor of glycogen synthase kinase 3 promotes a memory phenotype in IL13Rα2-CAR T cells and enhances their antitumor efficacy in preclinical HGG models (157). Several small molecule inhibitors have already been evaluated in humans and are effective and well tolerated (NCT00948259 and NCT02718911). Thus, utilizing these novel approaches to enhance long-term effector memory in CAR T–cell regimens for PBTs requires verification in the preclinical and clinical settings.

## Leveraging the Power of PBT Models to Improve CAR T–Cell Efficacy

Advancing CAR T–cell therapies for PBTs necessitates the use of adequate brain tumor models that that closely recapitulate human disease. Ideal models should be reproducible and easy to use, manipulate, and most importantly mimic the genetic, epigenetic, and phenotypic tumor heterogeneity and the TME of the human disease (158). Similar to the liquid and solid tumor animal models, brain tumor models are divided into patient derived orthotopic xenografts (PDOXs) and syngeneic models. Below we describe key characteristics and benefits of each model. Although humanized mouse models are gaining momentum and might be instrumental in future cell therapy studies, they are not discussed in this review.

### Patient-Derived Orthotopic Xenografts

PDOX models are generated by implanting cell suspensions of freshly isolated patient tumor tissues into the comparable tissue of origin in immunodeficient mice. The resultant tumors are closely representative of the original tumor heterogeneity in patients, including the stromal components, architecture, and biochemical interactions (159–162). Multiple studies have demonstrated that brain tumor PDOX models are transplantable and can be implanted into different brain locations (brain stem, cortex, thalamus, or cerebellum) (161, 163). After multiple passages of PDOX lines in immunocompromised mice, DNA and RNA sequencing of the resultant tumor xenografts revealed that most retain the heterogeneity of their matched patient sample (162). In addition, comparing surface-antigen profiling of PBT PDOX samples and matched patient samples showed that TAA expression is preserved in these models. In addition, the TAA expression is sustained throughout multiple passages (22). Therefore, PDOX models are ideal for target identification and validation and for evaluating the efficacy of novel cancer-directed therapies *in vivo*, especially since they retain their original TAA expression, chemical sensitivity, and drug resistance. However, PDOXs require the use of immunocompromised mice; this system constitutes a major limitation to evaluating the effects of immune contribution to treatment efficacy, resistance, and/or safety. Other disadvantages lie in the tumor latency; many PDOXs require a lengthy period (up to12 months) between implantation and development of tumors (164). This long latency makes it challenging to assess CAR T–cell therapy. In summary, PDOX models are an extremely valuable resource for evaluating CAR T–cell efficacy; however, additional models for validating key findings should be considered.

### Syngeneic pHGG Models

Syngeneic brain tumors can be implanted in immunocompetent mice to study the tumor’s biological interactions with the host’s immune system. Most syngeneic pHGGs are generated through platelet-derived growth factor (PDGF)-driven alterations (PDGFRA mutations or amplifications and/or PDGFB amplifications). These mutations are artificially introduced into neural stem cells (NSCs) (165). Implanting modified NSCs into neonatal mice forms supratentorial tumors that reproduce several features of pHGGs, including transcriptional and biological characteristics (165). Additionally, syngeneic DIPG models have been generated using combinations of mutations in *H3.3K27M* and *Pdgfra* and *p53* knockout in NSCs, which drive hindbrain tumorigenesis resulting in spontaneous DIPGs (166). These tumors recapitulate tumoral heterogeneity, the spontaneous nature of DIPGs, and the immune TME in DIPGs (166). Alternatively, introducing *H3K27M* mutations into human or murine embryonic stem cell–derived neural precursors, along with *PDGFRA-* and *TP53*-targeting mutations, produces transplantable and fast-growing DIPGs when implanted into SCID (severe combined immunodeficiency) mice (167, 168). Moreover, *in utero* electroporation into the brain stem of embryonic mice to insert the dominant-negative mutation of *p53*, *H3K27M*, and different combinations of *Pdgfb* amplification or *Pdgfra* mutation generates tumors with unique histopathologic and molecular features seen in human DMGs such as minimally disrupted BBBs (169). These models recapitulate the immune interactions and key features of the immune TME in pHGGs and will be very useful for studying CAR T–cell efficacy and safety.

The use of available animal models of PBTs will undoubtedly improve CAR T–cell evaluation and hasten the transition from preclinical to clinical testing. The challenge, however, is the availability of these models; not all investigators have access to them. In addition, some of the models require special handling, which will require additional training for CAR T–cell-focused laboratories. Adapting these models to the CAR T–cell testing pipeline will also pose some challenges. For example, some models can only be passaged *in vivo*, which complicates initial *in vitro* studies. Others might require special growth conditions for *in vitro* co-culture experiments that might not be compatible with T cells. On a positive note, these challenges might motivate productive collaborations between translational immunologists and brain tumor biologists that may result in more efficient efforts to generate safer, effective CAR T–cell therapies for PBTs.

## Conclusions and Future Perspectives

CAR T–cell therapy is a promising approach to treat PBTs. However, very few CAR T–cell studies have been done in a PBT setting. To advance the field and establish effective CAR T cells for PBTs, more studies are needed. Most importantly, studies must be done in PBT models, targeting PBT-specific antigens, and taking into account the tumor heterogeneity and unique features of the PBT TME. Although achieving this will be quite challenging, given the recent rapid advances in single-cell molecular approaches, preclinical model systems, and CAR design, it is not unreasonable to hope that it will be achievable in the near future.

## Author Contributions

DH, JI, and GK conceived, interpreted, and reviewed the literature. DH and GK conceptualized the research review, designed the figures, and wrote the manuscript. All authors contributed to the article and approved the submitted version.

## Funding

Funding was provided by NCI award K99CA256262 to DH and NIH/NINDS award R01NS121249 to GK.

## Conflict of Interest

GK has patent applications in the field of immunotherapy.

The remaining authors declare that the research was conducted in the absence of any commercial or financial relationships that could be construed as a potential conflict of interest.

## Publisher’s Note

All claims expressed in this article are solely those of the authors and do not necessarily represent those of their affiliated organizations, or those of the publisher, the editors and the reviewers. Any product that may be evaluated in this article, or claim that may be made by its manufacturer, is not guaranteed or endorsed by the publisher.
